# Levels of Trace Elements in the Aqueous Humor of Cataract Patients Measured by the Inductively Coupled Plasma Optical Emission Spectrometry

**DOI:** 10.3390/molecules24224127

**Published:** 2019-11-14

**Authors:** Joanna Dolar-Szczasny, Anna Święch, Jolanta Flieger, Małgorzata Tatarczak-Michalewska, Przemysław Niedzielski, Jędrzej Proch, Dariusz Majerek, Justyna Kawka, Jerzy Mackiewicz

**Affiliations:** 1 Department of Retina and Vitreo and nd Surgery, Medical University of Lublin, Chmielna 1, 20-079 Lublin, Poland; joannaszczasny@op.pl (J.D.-S.); jerzy.mackiewicz@umlub.pl (J.M.); 2 Department of Analytical Chemistry, Medical University of Lublin, Chodźki 4A, 20-093 Lublin, Poland; malgorzatatatarczakmichalewska@umlub.pl (M.T.-M.); justynakawka@umlub.pl (J.K.); 3 Faculty of Chemistry, Department of Analytical Chemistry, Adam Mickiewicz University in Poznań, 89B Umultowska Street, 61-614 Poznan, Poland; pnied@amu.edu.pl (P.N.); jed.proch@gmail.com (J.P.); 4 Department of Applied Mathematics, University of Technology, Nadbystrzycka 38D, 20-618 Lublin, Poland; d.majerek@pollub.pl

**Keywords:** trace elements, ICP-OES, cataract, the aqueous humor

## Abstract

Trace elements play an important role in the pathogenesis of several serious ophthalmological disorders, such as glaucoma, age-related macular degeneration (AMD), diabetic retinopathy, cataract, etc. This study aimed to measure alterations of chemical elements’ (67) levels in the aqueous humor of patients undergoing cataract surgery. The pilot study included 115 patients, (age 74 ± 7.27, female 64.35%, male 35.65%). The aqueous levels of elements were measured by the use of the inductively coupled plasma optical emission spectrometry (ICP-OES), quality controlled with certified standards. The classification of elements based on their concentration was achieved by hierarchical cluster analysis. This is the first screening study that quantifies over 60 elements which are present in the fluid from the anterior chamber of the eye of cataract patients. The obtained results can be suitable for understanding and identifying the causes that may play a role in the initiation and progression of lens opacity.

## 1. Introduction

A lens opacity that gets worse with age can finally lead to blindness [1]. Several indisputable causes of cataract formation have already been identified. It is known, for example, that the risk of lens opacity doubles every ten years [2]. Other risk factors are sex [3], diabetes [3], infrared (IR) and ultraviolet B (UV-B: 280–315 nm) radiation [4,5], and smoking [6,7]. However, there is still a need for new research to understand the causes of the formation and development of cataracts. Because cataract formation is strongly associated with age and cigarette smoking, which in turn lead to the accumulation of metal ions, many studies were devoted to analyzing the content of selected metals in human lenses or aqueous humor collected from patients undergoing cataract surgery.

Up to now, elevated levels of cadmium and selenium, as well as a downward trend in the copper content in the human lens, have been noted in relation to the age of patients [7]. A significant increase in aluminum and vanadium ions, a tendency to increase the content of chromium, nickel, and lead, as well as a tendency to decrease the concentration of copper ions, have been observed in smokers’ lenses compared to non-smokers [7]. There is a high probability that the observed changes in the metal ion content contribute to the formation of cataracts by inducing oxidative stress and modifying the structure and function of the extracellular matrix of the lens.

Without a doubt, metal ions are important for the human organism because many biological functions are dependent on their presence. The toxicity of metals occurs due to an essential metal overload as well as exposure to toxic heavy metals from different sources. Therefore, a metal ions scarcity, as well as excess, may provide numerous serious disorders. Toxic effects are caused either by heavy metal ions, such as mercury and lead, or by crucial essential metals if present in excess. Among the essential metals, the most notable include Fe, Co, Ni, Ca, Cu, Zn, and Cr. The deficiency of iron and cobalt provides anemia, the deficiency of copper leads, in turn, to brain and heart diseases, a lower level of zinc leads to growth retardation and skin changes, while a lower level of calcium leads to bone deterioration and a lower level of chromium reduces the glucose tolerance [8,9,10,11,12,13]. The role of trace metals is especially visible in the central nervous system (CNS), the haematopoietic system, the liver, the kidneys, etc. Exposure to heavy toxic metals has risen within the last 50 years as a result of an industrial increase. Metal ions such as zinc (Zn), iron (Fe), manganese (Mn), and copper (Cu) take part in the neurotransmitter synthesis at the synapse [14,15,16,17]. Therefore, concentrations of these metal ions must be tightly regulated in the nervous system [18]. Abnormal levels of metal ions can cause the progression of several neurodegenerative disorders, including Alzheimer’s disease [19,20,21,22], Parkinson’s disease [23], and Huntington’s disease [24]. There is some evidence that trace elements might play a role in the pathogenesis of ophthalmological neurodegenerative diseases such as glaucoma, the leading cause of irreversible blindness in the world or age-related macular degeneration (AMD) [25,26]. Junemann et al. [26] measured levels of selected metal ions in the aqueous humor of AMD-affected eyes by the use of Flow-Injection-Inductively-Coupled-Plasma-Mass-Spectrometry (FI-ICP-MS) and found significant alterations of trace element levels. It was observed that patients with AMD had significantly higher levels of cadmium, cobalt, iron, and zinc, as well as reduced level of copper, when compared with patients without AMD. In turn, for manganese and selenium, no significant differences were observed. Abnormal metal levels in the central visual system structures of the mouse model of glaucoma were observed by DeToma et al. [27]. The authors used inductively coupled plasma mass spectrometry (ICP-MS) to examine the levels of selected elements such as iron, copper, zinc, magnesium, manganese, and calcium in the retina of mice. ICP-MS experiments showed that mice with glaucoma had lower retinal Fe, Mg, Ca, Mn and Zn concentrations than pre-glaucomatous mice. More recently, the aqueous humor levels of cadmium, iron, manganese, cobalt, copper and zinc were measured in the aqueous humor samples of patients with primary open-angle glaucoma (POAG) and pseudo-exfoliation glaucoma (PEXG) by Flow-Injection-Inductively-Coupled-Plasma-Mass-Spectrometry (FI-ICP-MS) [28]. It was found that there were significant differences in the aqueous humor levels of zinc and iron between glaucoma and control patients. These findings support the initial hypothesis assumed by the authors that zinc and iron can play a role in the pathogenesis of open-angle glaucoma. In 1990, the determination of zinc and copper in the ophthalmic fluid of cataract and glaucoma patients was described [29]. A statistically significant negative relationship was observed between the concentrations of these elements in the aqueous humor of patients with cataracts and glaucoma. The concentrations of lead and cadmium were measured using an inductively coupled plasma-mass spectrometer in the fluids and tissues of human eyes [30]. It was observed that these toxic heavy metals are able to accumulate in human ocular tissues, especially in the retinal pigment epithelium and choroid.

Schmeling et al. [31] analyzed the aqueous humor and lens samples collected from 14 cataract patients to study the presence and concentration of selected heavy metals via the use of total reflection X-ray fluorescence spectrometry (TXRF). The data obtained show that the most commonly found metals in both analyzed media were chromium and manganese. Another metal found in the aqueous humor was a nickel.

Traditionally, the analysis of trace elements in biological samples is performed using widely available flame atomic absorption spectroscopy (FAAS) or graphite atomic absorption spectroscopy (GFAAS). The disadvantages of the mentioned methods are quite a large sample volume and an insufficient sensitivity, often requiring the enrichment of the sample by a targeted analyte in order to achieve appropriate detection limits. The quantification of elements at ultra-low levels is possible owing to more sophisticated mass spectrometry (MS) or atomic spectrometry, including atomic fluorescence spectrometry (AFS), atomic absorption spectrometry (AAS), and atomic emission spectrometry (AES) or optical emission spectrometry (OES). Unfortunately, atomic spectrometry techniques require aggressive sample preparation procedures that destroy the molecular structure of chemical species. For the speciation analysis, an on-line or off-line connection of two analytical techniques is required. There are many hyphenated techniques that are a combination of different separation methods, such as high-performance liquid chromatography (HPLC), size exclusion chromatography (SEC), gas chromatography (GC) or capillary electrophoresis (CE), with sensitive atomic spectrometry detectors for the separation, detection, identification and quantification of elemental species. It should be emphasized that hyphenated techniques absolutely require the use of improved sample preparation methods to preserve the species identity. To separate target species from complex biological matrices, different extraction methods are used, ranging from the oldest liquid-liquid extraction (LLE) to more advanced methods such as the ultrasonic micro-extraction of liquid-liquid dispersion (LLME), liquid-phase microextraction (LPME), solid-phase extraction (SPE), and solid-phase microextraction (SPME). The elimination of artifacts is possible due to the species-specific isotope dilution method. To eliminate non-spectral as well as spectral interferences caused by the presence of organic compounds or salts as components of solvents, used for elution, the concept of dispersed particle extraction (DPE) was introduced [32,33,34,35]. In this case, the analyte-containing sorbent is introduced as a specific element into an inductively coupled plasma optical emission spectrometry (ICP-OES) detection device. A further improvement of the repeatability, as well as the preconcentration of the sample, are possible thanks to the flow injection system (FI), which reduces the manual steps of the sample manipulation [36]. Recently, some review papers describing the speciation of trace elements by isolated or hyphenated techniques have been published [37,38,39]. Particularly the advances in the instrumentation, calibration strategies, and method development concerning ICP-OES are presented by Donati et al. [40]. In addition to these, there is a whole range of techniques useful for direct elementary speciation, such as X-rays, electron spectroscopy, electroanalysis, magnetic resonance spectroscopy, and nuclear spectroscopy techniques [41].

In the light of current achievements, the deficiency or excess of the various metals can help to study the etiology of the diseases at the molecular level and to find the appropriate remedy. Based on this knowledge, the aim of this study was to evaluate elements of aqueous humor in cataract patients. This is the first screening study that quantifies all metal ions appearing at a concentration not below 0.001 ppm in the aqueous humor of patients with lens opacity. More than sixty metal ions were determined in 115 donors aged between 55 and 94 years old. ICP-OES was chosen for the tests due to the small sample volumes available, the wide linear range for quantification, and the low detection limit. Furthermore, ICP-OES exhibits smaller interferences and a shorter time of analysis in comparison to the commonly applied ICP-MS. The observed dysregulation in the metal levels can help to understand the pathogenesis of cataracts. The collected database can be used as a reference for future research on the analysis of elements in the eye fluid.

## 2. Results

### 2.1. Element Content

Samples of fluid from the anterior chamber of the eye were obtained from patients undergoing cataract surgery: 115 patients between 55 and 94 years of age (age 74.94 ± 7.22, female 64.35%, male 35.65%). The levels of metals were measured by ICP-OES after an appropriate sample preparation. The measured values are collected in Table 1. Most elements showed substantial variations from patient to patient. The widest range refers to macroelements such as calcium (1062.194), sodium (5732.618), potassium (539.795), phosphorus (326.269), but also surprisingly cesium (350.0), which is highly toxic. The highest variability expressed by the CV was observed for lithium (1026.729), a fairly common element, but also for those that are dangerous such as arsenic (434.053) and cadmium (507.174). The large values of the standard deviations and wide ranges are not surprising considering the diversity of patients. There is also a significant group of elements that were not detected in most patients, which may point to their absence or occurrence at an undetectable level via the detection method that was used. This group includes transition elements: Ru, Pd, Os, Mn, Hg, Cd, Co, and V; post-transition metals like Sn; metalloids: B, Si, Ge, Sb; and rare earth elements: Tm, Tb, Sc, Pr, Lu, Ho, Ge, Gd, Eu, Er, Dy. On the other hand, the results show clear trends and provide initial information about the metal concentrations in the aqueous humor of cataract patients.

The main group, covering, i.e., sodium, potassium, and calcium, belongs to the so-called essential elements, and these elements occur at a concentration between 108.81 ppm for potassium and 2176.45 for sodium. The remaining elements occur at trace levels, though iron and zinc occur at about 1.692 and 0.357 ppm, respectively. All the other metals, as well as the three metalloids (silicon, arsenic, and selenium), occur only at ultra-trace levels, e.g., manganese and cobalt with about 0.001 and 0.039 ppm, respectively.

**Figure 1:**
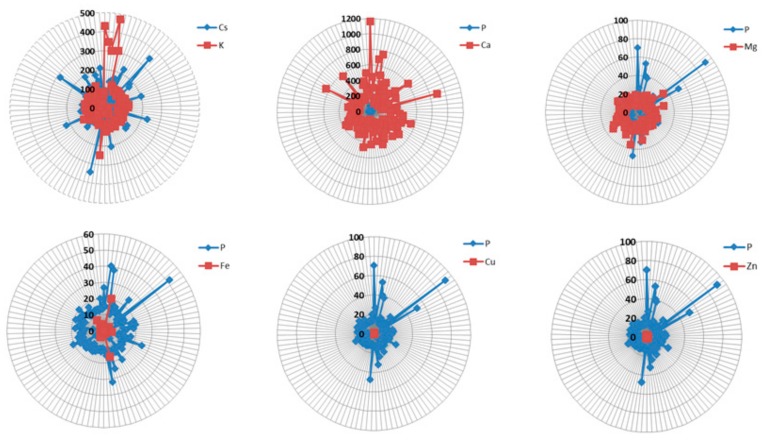
Radar charts show the content of selected elements in the aqueous humor of patients with cataracts.

Among the elements found in higher concentrations, the high cesium content is noteworthy. CsCl is sold as a supplement for alternative cancer therapy. However, its effectiveness has not been proven, and the collected evidence indicates a life-threatening toxicity. There is even a case of lethal toxicity after injection of a CsCl solution into a tumor [42]. The patient’s measured cesium level in the blood was 100,000 μg L^−1^ (reference range <10 μg L^−1^). However, death from an overdose of cesium supplements is very rare. High levels of cesium cause other serious problems, such as cardiac disorders, unconsciousness, convulsions and electrolyte disturbances, including sodium and potassium. Health hazard arises from the chemical similarity of Cs to potassium. The danger of poisoning comes primarily from environmental pollution. In areas of eastern Poland where radioactive contamination of the Chernobyl nuclear power plant occurred on April 28, 1986, cesium-137 contamination of 3–8 kBq/m^2^ was found [43]. The comparison presented in Figure 1 shows that the content of competitive potassium is relatively stable and generally higher than the cesium content in the ophthalmic fluid; however, in 20% of cases, an elevated cesium level was observed, which indicates the accumulation of this element. Given the advanced age of patients and the place of residence (areas of eastern Poland affected by cesium contamination), this seems to be justified.

Phosphorus belongs to macro elements, and its content in the body is greater than 0.01% of the body weight, while the daily demand exceeds 100 mg/d. Phosphorus is crucial for bone and tooth health. Phosphorus performs its functions in the body provided that there is a balance between it and the amount of calcium. A possible phosphorus deficiency reduces calcium absorption. In turn, phosphorus overdosing causes leaching of calcium from the body. It is very important that the balance between these elements is maintained in the human body. When analyzing calcium and phosphorus levels, it can be seen that calcium levels are much higher compared to phosphorus. It should be remembered that the level of phosphorus affects the absorption and levels of some microelements in body fluids such as magnesium, iron, zinc, and copper. Interactions between iron, calcium, phosphorus, magnesium, copper, and zinc were studied in iron-deficient rats [44]. It has been found that iron deficiency is associated with an increase in the absorption of phosphorus and magnesium. 

In the group of elements present at a trace level, the high content of aluminum, lead, tellurium, and thallium is noteworthy. Until now, no one has studied the level of these elements, although their concentrations are much higher than, e.g., copper, chromium, selenium or zinc, which was previously determined in the aqueous humor. So far, high levels of aluminum (2.3%) have been found in lenses with cataract in smokers [7]. This is the first study showing a high Al content in ophthalmic fluid in people with cataracts. Because Al is involved in neurodegenerative processes, this is a clinically relevant observation. As has been shown so far, a chronic exposure to Al causes its accumulation in various tissues [45,46,47,48,49,50,51]. It has been proven that even low doses of aluminum are neurotoxic, induce inflammatory processes, and adversely affect cognitive functions and morphological changes in the central nervous system. It is very likely that the increased accumulation of Al is due to the general increased oral exposure to this element. It has already been confirmed that silicon can have a protective effect in the case of aluminum contamination [51,52,53]. An increased intake of silicon probably mobilizes various forms of aluminum in vivo and reduces its storage in tissues. There is evidence that silicon reduces the uptake of aluminum in the intestine. High levels of aluminum (mean value: 2.427 ppm) were detected in the aqueous humor of all patients. The fairly low coefficient of variation for the occurrence of this element in the studied group (CV = 69) confirms the fact that patients are commonly exposed to this element. The co-occurrence of silicon in less than half of the patients (43.3%) seems to confirm Si–Al interactions in tissues in vivo.

All of the tested samples contained high thallium levels within 0–16.9 ppm. Thallium is a highly toxic element, comparable to lead, cadmium, and mercury. Thallium (III) compounds show a similar toxicity to mercury (II) compounds, and less toxic thallium (I) compounds are up to 10 times more toxic than lead (II) compounds. Tal has mutagenic, carcinogenic and teratogenic effects, among others. The accumulation of this element causes gastrointestinal ulcers, alopecia, and polyneuropathy. Thallium dispersion in the environment is mainly due to the activity of industry [54,55,56]. The main anthropogenic sources of thallium are solid emissions and wastes from coal, iron, non-ferrous mining, and metallurgy or cement production processes. It can get into surface waters, soils, plants and, as a consequence, into the digestive system. Hair has already been tested for thallium content [57,58], as well as blood and urine [59]. It turns out that highly-soluble thallium (I) compounds are easily absorbed, even through skin [60].

Much is known about lead toxicity [61], whose measured concentration ranges between 0–8.8 ppm; surprisingly little information is available on the presence and concentration of other toxic metals in the eye tissue and their effects on both sight and sharpness. To date, there are no studies that show such high contents of another toxic element, namely tellurium, in the ophthalmic fluid. The measured level in patients with cataracts ranges from 0.2 to 8.4 ppm. The human body absorbs tellurium with food and it accumulates in the bones, liver, and spleen. Due to human health hazards, tellurium has been classified in the Regulation of the European Parliament and of the Council (EC) No. 1272/2008w as a substance showing acute inhalation toxicity, causing serious eye damage/eye irritation, and toxic to internal organs and reproduction. Tellurium in the tested material occurs at a level comparable to lead, thallium, and aluminum. The accumulation of these toxic elements in the aqueous humor in people with cataracts is of clinical importance and sheds new light on the causes of this disease.

### 2.2. Statistical Analysis

The aim of the statistical analysis is to divide all chemical elements in the eye fluid of patients undergoing cataract surgery, based on their concentration. Using a statistical analysis, a non-arbitrary division was achieved. Due to the nature of the data, unsupervised machine learning methods were used [62]. The division into homogeneous groups was applied using pairwise comparisons [63] and a cluster analysis [64]. 

The distributions of the chemical elements in the raw data were characterized by a high right-hand side asymmetry. This phenomenon is widely known. Asymmetry is affected by the fact that many people have low levels of chemical element concentration. The lowest asymmetry is for Te (A = 1.07) and the highest for Li (A = 10.7).

**Figure 2:**
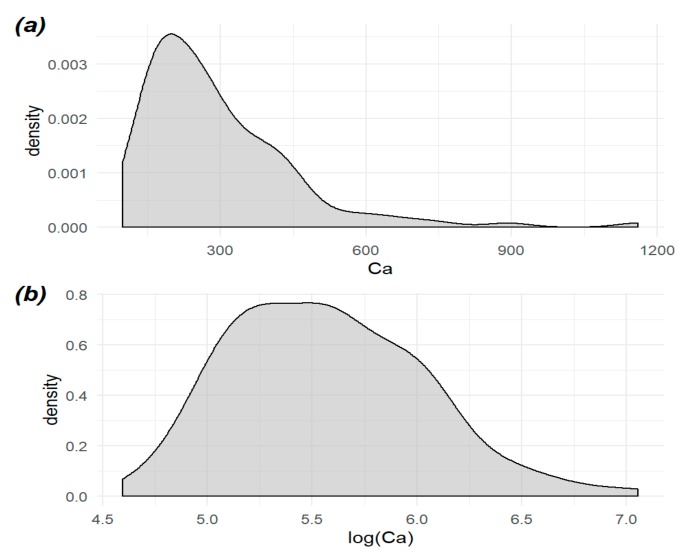
Distribution of the (**a**) raw and (**b**) transformed concentration of Ca. The transformed version is more symmetric.

There were two reasons why the transformation of the raw concentration of elements was needed. First, all of the earlier mentioned statistical methods need to resemble a symmetric distribution. Second, the division of the elements obtained from the raw data is not interesting. Using a logarithmic transformation of the elements’ concentration, a more interesting partition was obtained. Figure 2 illustrates the changes in the distribution between the transformed and raw concentrations of Ca [65]. 

#### 2.2.1. Division Based on Pairwise Comparisons

The idea of dividing elements into groups is based on the idea that elements that have similar levels of concentration should be in one group. However, large differences in the concentration of particular elements make it difficult to discriminate between them.

**Figure 3:**
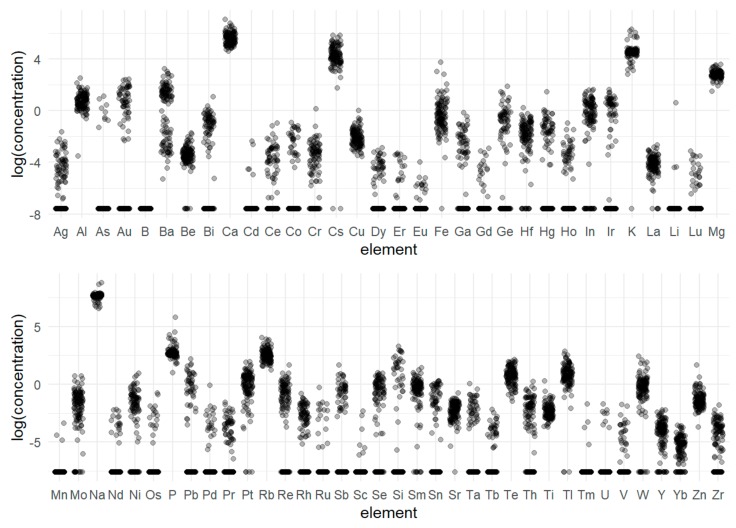
The distributions of the transformed concentrations of elements.

Figure 3 shows that there are differences between the concentrations of elements. A first attempt to group them in view of the concentration was based on pairwise comparisons [66]. An ANOVA test confirmed that the differences in the means of the concentrations between the elements are significant (F = 333.93 and p = 0). However, a post hoc test [66] failed in the creation of groups because almost all elements were characterized by a bimodal distribution, which means that there are two groups of observations for a particular element, one with a near-zero concentration and a second with a higher concentration (i.e., see Au in Figure 3). The groups thus obtained are superimposed on each other.

#### 2.2.2. Division Based on Hierarchical Cluster Analysis

The second approach was based on a hierarchical cluster analysis. This method aims to divide the observations into k homogeneous groups, characterized by similar levels of characteristics describing them, while maximizing the dissimilarities between the groups. The similarity is measured by a Euclidean metric. In each step of the agglomerative hierarchical approach, an observation or a cluster of observations is merged into another cluster (the two closest clusters or observations are merged into a single new cluster). In this process, the number of clusters shrinks, and the clusters themselves grow larger. To measure the similarities between clusters, Ward’s method was used, which uses intra-group and inter-group variability [67].

Using this method for each observation separately, you can obtain a division in which the same element will be in two different groups because its concentration for one patient will be higher and lower for the other. Therefore, averaged logarithmized concentrations of individual elements were first used for all patients. As a result, a hard division of elements was obtained, which means that each element belongs to only one group.

**Figure 4:**
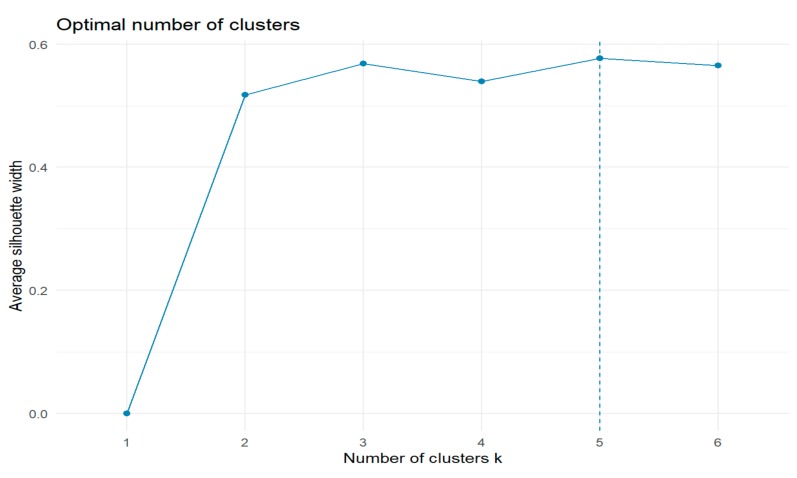
Silhouette criterion plot.

The results of the hierarchical clustering are usually expressed by a dendrogram, on the basis of which it is estimated how many groups should be formed. There are also other methods for assessing the number of clusters, and for thus determining the distribution of observations into groups. One of them is the silhouette method (Figure 4) [68], which indicates that the division into 5 clusters will be good. However, both the silhouette criterion and dendrogram show that a division into 3 groups is also reasonable.

**Figure 5:**
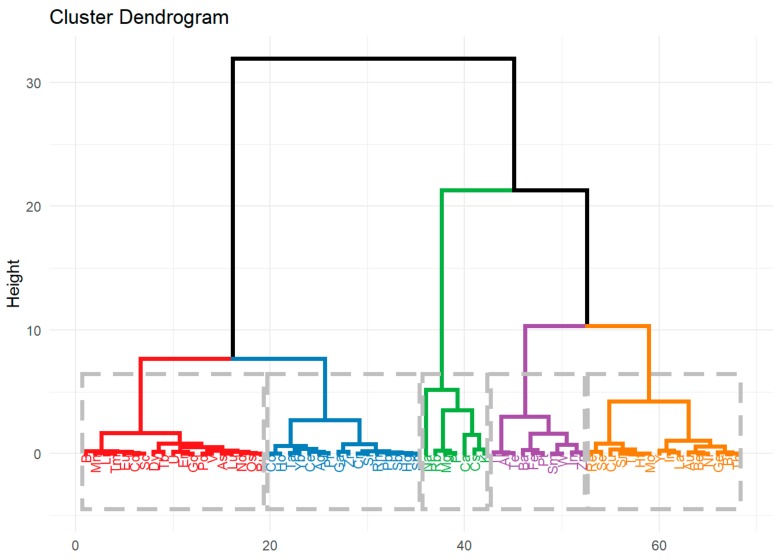
Dendrogram of the averaged logarithm of the concentrations of elements.

Based on hierarchical cluster analysis (Figure 5), the following division was obtained:

- Cluster 1. - Ag, Ce, Co, Cr, Ga, Hg, Ho, Pb, Pr, Rh, Sb, Si, Sn, Ta, Yb, Zr,
- Cluster 2. - Al, Ba, Fe, In, Pt, Sm, Te, Tl, W, Zn,
- Cluster 3. - As, B, Cd, Dy, Er, Eu, Gd, Li, Lu, Mn, Nd, Os, Pd, Ru, Sc, Tb, Tm, U, V,
- Cluster 4. - Au, Be, Bi, Cu, Ge, Hf, Ir, La, Mo, Ni, Re, Se, Sr, Th, Ti, Y,
- Cluster 5. - Ca, Cs, K, Mg, Na, P, Rb.

It can be seen that the 5th cluster consists of elements of the highest concentration (see Table 2). Cluster 2 is made of elements with the second-highest concentration. The lowest element concentrations are in cluster 3 because both central tendency measures (median and mean) are minimal. The increasing order of the groups in view of the element concentrations is as follows: cluster 3, cluster 1, cluster 4, cluster 2, and cluster 5. The least homogenous group is cluster 5 because it has the largest variance. Moreover, as the level of concentration increases, its variance also increases.

**Figure 6:**
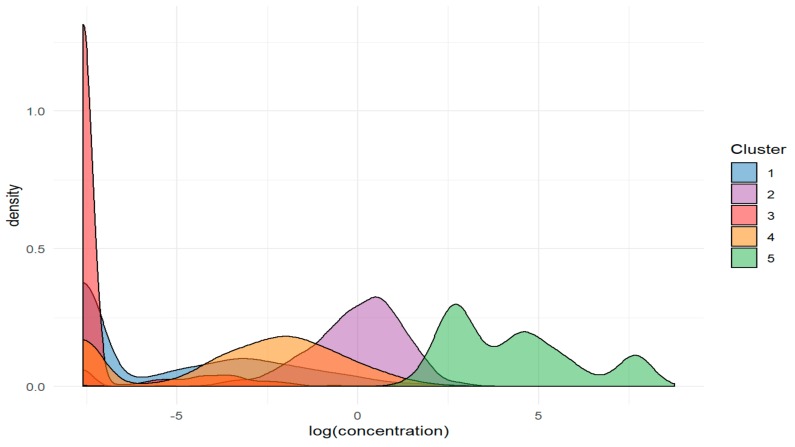
The densities of the element concentration in clusters.

The distribution of the averaged logarithm of the concentrations of elements can be described by the densities (Figure 6). Even after the aggregation of the logarithmized concentrations, the distributions are bimodal. Quite similar results are obtained if 3 clusters are chosen. In this case, clusters 1 and 3 are merged into one group, and clusters 2 and 4 are merged into another one.

## 3. Materials and Methods

### 3.1. Subjects

The research material consisted of the aqueous humor from the anterior chamber of the eye taken from patients undergoing cataract surgery in The Department of Retina and Vitreous Surgery at the Medical University of Lublin in Poland. The study was approved by the Local Bioethical Committee of the Medical University of Lublin (approval No. KE-0254/271/2019). The work has been carried out in accordance with The Code of Ethics of the World Medical Association (Declaration of Helsinki) for experiments involving humans. The samples were collected from 115 subjects (41 male, 74 female) at the beginning of the cataract extraction surgery.

### 3.2. Sample Preparation Procedure

The samples were stored in 1.5 mL polypropylene tubes at −80 °C until analysis. A wet mineralization of each sample was performed via the addition of 2 mL of 65% suprapur nitric acid HNO_3_ (Merck, Darmstadt, Germany), followed by heating to 180 °C in close Teflon containers in the microwave mineralization system Mars 6 (CEM, Matthews, NC, USA). Finally, the samples were diluted to 10 mL by ultrapure water obtained in the purification system Milli-Q (Millipore, Darmstadt, Germany).

### 3.3. Analytical Procedures

The inductively coupled plasma optical emission spectrometer Agilent 5110 ICP-OES (Agilent, Santa Clara, CA, USA) was employed for an elemental analysis. In doing so, the radio frequency (RF) power was 1.2 kW, the nebulizer gas flow was 0.7 L min^−1^, the auxiliary gas flow was 1.0 L min^−1^, the plasma gas flow was 12.0 L min^−1^, the charge coupled device (CCD) temperature was −40 °C, the viewing height for the radial plasma observation was 8 mm, while the accusation time was 5 s. The analysis was repeated three times. ICP commercial analytical standards (Romil, Cambridge, UK) were used for the calibration. The detection limits (LOD) were determined through 3-sigma criteria and were on the level of 0.001 (ppm) wet weight (*w*/*w*) for all elements determined. The uncertainty for the complete analytical process (including the sample preparation) was at the level of 20%. The traceability was assessed by a standard addition procedure. A recovery of 80–120% was considered acceptable for all the determined elements.

### 3.4. Statistical Analysis

All calculations and illustrations were done using statistical environment R Core Team 2019 [69] with necessary packages [70,71,72,73].

## 4. Conclusions

The role of metals in physiological and pathological processes has been of interest to scientists for years. According to recent works, metals also contribute to the pathogenesis and development of eye diseases. Until now, the morphological structures of the eyes were examined only for the content of selected metals, such as Co, Cd, Zn, Fe, Cu, Mn, and Se. This work is the first study that classifies all metals present in concentrations exceeding the detection limit of the ICP-OES determination method, i.e., 0.001 ppm, present in the fluid from the anterior chamber of the eye of patients undergoing cataract surgery. The analysis showed the existence of a statistically significant series of metals present at various concentration levels. The classification of elements was made by a hierarchical cluster analysis. The existence of 5 independent clusters was statistically justified. In the group of elements found at high concentration levels (cluster 5), the results obtained for phosphorus are noteworthy. While, the other elements belonging to this group are characterized by a fairly small coefficient of variation (29.572–72.705), phosphorus has a coefficient about twice as large, i.e., 158,792. There is, therefore, a significant variation in the levels of this element in the patient population being studied. In future research, therefore, particular attention should be paid to this element due to its effect on the calcium distribution and absorption of microelements in body fluids.

This study showed for the first time that patients with cataracts are characterized by elevated levels of very toxic elements in the anterior chamber fluid. Cluster number 2 contains, e.g., thallium and tellurium. The fifth cluster of elements with the highest concentration contains toxic cesium. Considering the fact that these metals, along with lead or aluminum (of known prevalence and toxicity) have a strong neurodegenerative potential, the observations made are clinically relevant. Among the toxic elements, the variability of the levels of Al (CV = 69.146), Cs (CV = 73.205), Te (CV = 68.316) and Tl (CV = 90.629) in the population is noteworthy, while the variability of Pb (CV = 220.678), Cd (507.174), and Bi (CV = 146.727) is definitely higher. This result illustrates the diversity of patients’ exposure to poisoning by these elements. Low levels of silicon with recognized protection properties against aluminum contamination and disturbed proportions of Cs–K, P–Ca, P–Mg, P–Fe, P–Cu, P–Zn were also found. The performed tests confirm the increased exposure of patients with cataracts to toxic elements.

## Figures and Tables

**Table 1:** Element concentrations’ descriptive statistics.

Element/Wavelength [nm]	BDL (n)	ADL (n)	Mean [ppm]	SD	Median [ppm]	Min. [ppm]	Max [ppm]	Range [ppm]	CV
Ag/328.068	53	62	0.014	0.027	0.003	0.000	0.191	0.191	193.898
Al/396.152	0	115	2.427	1.678	1.948	0.029	12.449	12.420	69.146
As/188.980	106	9	0.097	0.419	0.000	0.000	3.071	3.071	434.053
Au/197.742	59	56	1.658	2.640	0.000	0.000	11.284	11.284	159.214
B/249.772	115	0	0.000	0.000	0.000	0.000	0.000	0.000	0.000
Ba/455.403	0	115	3.547	4.551	2.869	0.005	25.461	25.456	128.288
Be/313.042	7	108	0.038	0.029	0.033	0.000	0.176	0.175	76.811
Bi/223.061	49	66	0.276	0.404	0.090	0.000	3.003	3.002	146.727
Ca/422.673	0	115	294.508	162.330	259.183	98.981	1161.175	1062.194	55.119
Cd/214.439	110	5	0.002	0.011	0.000	0.000	0.094	0.094	507.174
Ce/446.021	60	55	0.023	0.052	0.000	0.000	0.382	0.381	224.203
Co/238.892	82	33	0.039	0.082	0.000	0.000	0.397	0.397	210.387
Cr/267.716	38	77	0.041	0.114	0.015	0.000	1.171	1.170	280.103
Cs/697.327	2	113	89.742	65.695	71.684	0.000	350.000	350.000	73.205
Cu/327.395	0	115	0.154	0.128	0.117	0.029	1.021	0.991	83.088
Dy/364.540	75	40	0.006	0.011	0.000	0.000	0.057	0.057	171.710
Er/349.910	97	18	0.003	0.008	0.000	0.000	0.037	0.037	256.549
Eu/420.504	100	14	0.001	0.002	0.000	0.000	0.019	0.018	199.784
Fe/238.204	1	114	1.692	4.649	0.593	0.000	42.727	42.726	274.763
Ga/294.363	63	52	0.069	0.133	0.000	0.000	0.847	0.846	193.997
Gd/342.246	96	19	0.003	0.008	0.000	0.000	0.055	0.054	274.318
Ge/209.426	54	61	0.477	0.957	0.048	0.000	6.553	6.553	200.517
Hf/264.141	12	103	0.223	0.204	0.166	0.000	1.274	1.274	91.585
Hg/194.164	61	54	0.190	0.456	0.000	0.000	4.303	4.302	239.589
Ho/348.484	74	41	0.021	0.051	0.000	0.000	0.382	0.381	238.256
In/230.606	22	93	1.119	1.138	0.741	0.000	5.129	5.128	101.660
Ir/205.116	63	51	0.727	1.100	0.000	0.000	5.098	5.098	151.379
K/769.897	1	114	108.810	79.111	94.219	0.000	539.796	539.795	72.705
La/398.852	5	110	0.018	0.012	0.016	0.000	0.070	0.069	66.576
Li/670.783	112	3	0.017	0.171	0.000	0.000	1.837	1.836	1026.729
Lu/307.760	90	25	0.003	0.007	0.000	0.000	0.044	0.044	234.920
Mg/285.213	0	115	16.959	5.115	16.041	4.525	35.594	31.069	30.159
Mn/257.610	112	3	0.001	0.003	0.000	0.000	0.034	0.034	351.520
Mo/202.032	13	102	0.283	0.361	0.188	0.000	2.042	2.042	127.379
Na/588.995	0	115	2176.450	643.624	2199.520	697.710	6430.328	5732.618	29.572
Nd/406.108	97	18	0.007	0.020	0.000	0.000	0.113	0.113	278.466
Ni/231.604	40	75	0.250	0.406	0.125	0.000	2.533	2.533	162.504
Os/225.585	98	17	0.017	0.063	0.000	0.000	0.472	0.472	379.535
P/213.618	0	115	19.734	31.336	14.025	2.742	329.010	326.269	158.792
Pb/220.353	71	44	0.615	1.358	0.000	0.000	8.833	8.832	220.678
Pd/340.458	93	22	0.017	0.086	0.000	0.000	0.893	0.892	507.677
Pr/417.939	65	50	0.018	0.035	0.000	0.000	0.207	0.207	189.612
Pt/203.646	4	111	1.418	1.293	1.083	0.000	7.185	7.185	91.241
Rb/780.026	0	115	14.551	9.167	12.384	3.527	56.826	53.299	63.001
Re/197.248	33	82	0.517	0.710	0.283	0.000	5.172	5.171	137.156
Rh/343.488	46	69	0.062	0.083	0.029	0.000	0.438	0.438	134.481
Ru/240.272	98	17	0.019	0.081	0.000	0.000	0.768	0.768	420.936
Sb/206.834	68	47	0.341	0.719	0.000	0.000	5.407	5.407	211.004
Sc/361.383	108	7	0.002	0.011	0.000	0.000	0.100	0.099	506.182
Se/196.026	38	77	0.609	0.697	0.418	0.000	2.687	2.687	114.311
Si/288.158	78	37	1.986	4.690	0.000	0.000	26.102	26.102	236.114
Sm/442.434	8	107	0.840	0.685	0.746	0.000	4.253	4.252	81.588
Sn/283.998	64	51	0.227	0.368	0.000	0.000	1.340	1.340	161.961
Sr/460.733	1	114	0.122	0.076	0.112	0.000	0.478	0.478	62.245
Ta/268.517	72	43	0.072	0.150	0.000	0.000	1.044	1.043	208.023
Tb/350.914	85	30	0.008	0.017	0.000	0.000	0.114	0.114	225.447
Te/214.282	0	115	2.672	1.826	2.159	0.236	8.404	8.168	68.316
Th/283.730	38	77	0.137	0.198	0.061	0.000	1.294	1.294	144.333
Ti/336.122	0	115	0.122	0.140	0.090	0.025	1.350	1.325	114.598
Tl/190.794	3	112	3.101	2.810	2.081	0.000	16.943	16.943	90.629
Tm/336.261	111	4	0.003	0.017	0.000	0.000	0.180	0.180	652.877
U/385.957	107	8	0.006	0.024	0.000	0.000	0.184	0.183	399.184
V/292.401	88	27	0.007	0.023	0.000	0.000	0.172	0.171	316.212
W/207.912	11	104	1.148	1.602	0.768	0.000	11.296	11.296	139.611
Y/361.104	15	100	0.024	0.023	0.018	0.000	0.109	0.108	97.013
Yb/328.937	20	93	0.006	0.006	0.005	0.000	0.029	0.028	96.014
Zn/213.857	0	115	0.357	0.520	0.234	0.025	5.163	5.138	145.568
Zr/343.823	35	80	0.020	0.025	0.012	0.000	0.162	0.162	128.335

Abbreviations: BDL - Below detection level, ADL - Above detection level, n - Number of subjects, Range - (max - min), SD - standard deviation.

**Table 2:** Element concentrations [ppm] descriptive statistics.

Group	Min.	Median	Mean	Max.	SD
1 2 3 4 5	0 0 0 0 0	0.000 1.071 0.000 0.072 62.918	0.234 1.832 0.012 0.352 388.679	26.102 42.727 3.071 11.284 6430.328	1.329 2.685 0.111 0.904 778.327

## References

[1] Pascolini D., Mariotti S.P. (2012). Global estimates of visual impairment: 2010. Br. J. Ophthalmol..

[2] McCarty C.A., Keeffe J.E., Taylor H.R. (1999). The need for cataract surgery: Projections based on lens opacity, visual acuity, and personal concern. Br. J. Ophthalmol..

[3] Chang J.R., Koo E., Agrón E., Hallak J., Clemons T., Azar D., Sperduto R.D., Ferris F.L., Chew E.Y. (2011). Risk factors associated with incident cataract and cataract surgery in the Age-related Eye Disease Study (AREDS): AREDS report number 32. Ophthalmology.

[4] Lydahl E. (1984). Infrared radiation and cataract. Acta Ophthalmol. Suppl..

[5] Brian G., Taylor H. (2001). Cataract blindness--challenges for the 21st century. Bull. World Health Organ..

[6] Ye J., He J., Wang C., Wu H., Shi X., Zhang H., Xie J., Lee S.Y. (2012). Smoking and risk of age-related cataract: A meta-analysis. Invest. Ophthalmol. Vis. Sci..

[7] Langford-Smith A., Tilakaratna V., Lythgoe P.R., Clark S.J., Bishop P.N., Day A.J. (2016). Age and Smoking Related Changes in Metal Ion Levels in Human Lens: Implications for Cataract Formation. PLoS ONE.

[8] Sigel H. (1982). Metal Ions in Biological Systems: Inorganic Drugs in Deficiency and Disease.

[9] Holm R.H., Kennepohl P., Solomon E.I. (1996). Structural and Functional Aspects of Metal Sites in Biology. Chem. Rev..

[10] Lippard S.J., Berg J.M. (1994). Principles of Bioinorganic Chemistry.

[11] Cowan J.A. (1994). Inorganic Biochemistry/An Introduction.

[12] Burtis C.A., Ashwood R.E., Bruns D.E. (1994). Tietz Textbook of Clinical Chemistry.

[13] Gupta S.P. (2018). Roles of metals in human health. MOJ Bioorg. Org. Chem..

[14] Sourkes T.L. (1972). Influence of specific nutrients on catecholamine synthesis and metabolism. Pharmacol Rev..

[15] Paoletti P., Vergnano A.M., Barbour B., Casado M. (2009). Zinc at glutamatergic synapses. Neuroscience.

[16] Corona C., Pensalfini A., Frazzini V., Sensi S.L. (2011). New therapeutic targets in Alzheimer’s disease: Brain deregulation of calcium and zinc. Cell Death Dis..

[17] Südhof T.C. (2012). Calcium control of neurotransmitter release. Cold Spring Harb Perspect Biol..

[18] Sigel A., Sigel H., Sigel R.K.O. (2006). Neurodegenerative Diseases and Metal Ions: Metal Ions in the Life Sciences.

[19] DeToma A.S., Salamekh S., Ramamoorthy A., Lim M.H. (2012). Misfolded proteins in Alzheimer’s disease and type II diabetes. Chem Soc Rev..

[20] Pithadia A.S., Lim M.H. (2012). Metal-associated amyloid-β species in Alzheimer’s disease. Curr. Opin. Chem. Biol..

[21] Kepp K.P. (2012). Bioinorganic chemistry of Alzheimer’s disease. Chem. Rev..

[22] Savelieff M.G., Lee S., Liu Y., Lim M.H. (2013). Untangling amyloid-β, tau, and metals in Alzheimer’s disease. ACS Chem. Biol..

[23] Bisaglia M., Tessari I., Mammi S., Bubacco L. (2009). Interaction between alpha-synuclein and metal ions, still looking for a role in the pathogenesis of Parkinson’s disease. Neuromolecular Med..

[24] Dexter D.T., Jenner P., Schapira A.H., Marsden C.D. (1992). Alterations in levels of iron, ferritin, and other trace metals in neurodegenerative diseases affecting the basal ganglia. The Royal Kings and Queens Parkinson’s Disease Research Group. Ann. Neurol..

[25] Kass D.K., Heuer E.J., Higginbotham C.A., Johnson J.L., Keltner J.P., Parrish R.K., Wilson M.R., Gordon M.O. (2002). The ocular hypertension treatment study: A randomized trial determines that topical ocular hypotensive medication delays or prevents the onset of primary open-angle glaucoma. Arch. Ophthalmol..

[26] Junemann A.G.M., Stopa P., Michalke B., Chaudhri A., Reulbach U., Cord Huchzermeyer C., Schlotzer-Schrehardt U., Kruse F.E., Zrenner E., Rejdak R. (2013). Levels of Aqueous Humor Trace Elements in Patients with Non-Exsudative Age-related Macular Degeneration: A Case-control Study. PLoS ONE.

[27] DeToma A.S., Dengler-Crish C.M., Deb A., Braymer J.J., Penner-Hahn J.E., van der Schyf C.J., Lim M.H., Crish S.D. (2014). Abnormal metal levels in the primary visual pathway of the DBA/2J mouse model of glaucoma. Biometals.

[28] Hohberger B., Chaudhri M.A., Michalke B., Lucio M., Nowomiejska K., Schlötzer-Schrehardt U., Grieb P., Rejdak R., Jünemann A.G.M. (2018). Levels of aqueous humor trace elements in patients with open-angle Glaucoma. J. Trace Elem. Med. Biol..

[29] Akyol N., Deger O., Keha E.E., Kilic S. (1990). Aqueous-humorand serum zinc and copper concentrations of patients with glaucoma and cataract. Br. J. Ophthalmol..

[30] Erie J.C., Butz J.A., Good J.A., Erie E.A., Burritt M.F., Cameron J.D. (2005). Heavy Metal Concentrations in Human Eyes. Am. J. Ophthalmol..

[31] Schmeling M., Gaynes B.I., Tidow-Kebritchi S. (2014). Heavy metal analysis in lens and aqueous humor of cataract patients by total reflection X-ray fluorescence spectrometry. Powder Diffr..

[32] Bauer G., Neouze M.A., Limbeck A. (2013). Dispersed particle extraction — A new procedure for trace element enrichment from natural aqueous samples with subsequent ICP-OES analysis. Talanta.

[33] Janski R., Neouze M.A., Limbeck A. (2014). Determination of rare earth elements in saline matrices using dispersed particle extraction and inductively coupled plasma mass spectrometry. Rapid Commun. Mass Spectrom..

[34] Nischkauer W., Neouze M.A., Vanhaecke F., Limbeck A. (2015). Extraction and pre-concentration of platinum and palladium from microwave-digested road dust via ion exchanging mesoporous silica microparticles prior to their quantification by quadrupole ICP-MS. Microchim. Acta.

[35] Hosseinzadegan S., Nischkauer W., Bica K., Limbeck A. (2016). Bioparticles coated with an ionic liquid for the pre-concentration of rare earth elements from microwave-digested tea samples and the subsequent quantification by ETV-ICP-OES. Anal. Methods.

[36] Hosseinzadegan S., Nischkauer W., Bica K., Limbeck A. (2019). FI-ICP-OES determination of Pb in drinking water after pre-concentration using magnetic nanoparticles coated with ionic liquid. Microchem. J..

[37] Yu X., Liu C., Guo Y., Deng T. (2019). Speciation Analysis of Trace Arsenic, Mercury, Selenium and Antimony in Environmental and Biological Samples Based on Hyphenated Techniques. Molecules.

[38] Marcinkowska M., Barałkiewicz D. (2016). Multielemental speciation analysis by advanced hyphenated technique – HPLC/ICP-MS: A review. Talanta.

[39] Sperling M., Kars U. (2018). Advances in speciation techniques and methodology. Trends Anal. Chem..

[40] Donati G.L., Amais R.S., Williams C.B. (2017). Recent advances in inductively coupled plasma optical emission spectrometry. J. Anal. At. Spectrom..

[41] McSheehy S., Sperling M. (2009). Hyphenated ICP-MS Techniques for Speciation Analysis. Spectroscopy.

[42] Sessions D., Heard K., Kosnett M. (2013). Fatal Cesium Chloride Toxicity After Alternative Cancer Treatment. J. Altern. Complement. Med..

[43] Jaworowski Z. (1996). Jak to z Czarnobylem było. Wiedza i Życie.

[44] Gómez-Ayala A.E., Lisbona F., López-Aliaga I., Pallarés I., Barrionuevo M., Hartiti S., Rodríguez-Matas M.C., Campos M.S. (1998). The absorption of iron, calcium, phosphorus, magnesium, copper and zinc in the jejunum-ileum of control and iron-deficient rats. Lab Anim..

[45] Chaussidon M., Netter P., Kessler M., Membre H., Fener P., Delons S., Albarède F. (1993). Dialysis-associated arthropathy: Secondary ion mass spectrometry evidence of aluminum silicate in β-microglobulin amyloid synovial tissue and articular cartilage. Nephron.

[46] Muma N.A., Singer S.M. (1996). Aluminum-induced neuropathology: Transient changes in microtubule-associated proteins. Neurotoxicol. Teratol..

[47] Murayama H., Shin R.W., Higuchi J., Shibuya S., Muramoto T., Kitamoto T. (1999). Interaction of aluminum with PHFtau in Alzheimer’s disease neurofibrillary degeneration evidenced by desferrioxamine-assisted chelating autoclave method. Am. J. Pathol..

[48] Uversky V.N., Fink A.L. (2001). Metal-triggered structural transformations, aggregation, and fibrillation of human α-synuclein: A possible molecular link between parkinson’s disease and heavy metal exposure. J. Biol. Chem..

[49] Khan A., Ashcroft A.E., Korchazhkina O.V., Exley C. (2004). Metal-mediated formation of fibrillar ABri amyloid. J. Inorg. Biochem..

[50] Rodella L.F., Ricci F., Borsani E., Stacchiotti A., Foglio E., Favero G., Rezzani R., Mariani C., Bianchi1 R. (2008). Aluminium exposure induces Alzheimer’s disease-like histopathological alterations in mouse brain. Histol. Histopathol..

[51] Grochowski C., Blicharska E., Bogucki J., Proch J., Mierzwińska A., Baj J., Litak J., Podkowiński A., Flieger J., Teresiński G. (2019). Increased Aluminum Content in Certain Brain Structures is Correlated with Higher Silicon Concentration in Alcoholic Use Disorder. Molecules.

[52] Exley C., Chappell J.S., Birchall J.D. (1991). A mechanism for acute aluminium toxicity in fish. J. Theor. Biol..

[53] Edwardson J.A., Moore P.B., Ferrier I.N., Lilley J.S., Newton G.W.A., Barker J., Templar J., Day J.P. (1993). Effect of silicon on gastrointestinal absorption of aluminium. Lancet.

[54] Vanek A., Chrastny V., Komarek V., Penizek V., Teper L., Cabala J., Drabek O. (2013). Geochemical position of thallium in soils from a smelter-impacted area. J. Geochem. Explor..

[55] Lukaszewski Z., Jakubowska M., Zembrzuski W., Pasieczna A., Karbowska B. (2010). Flow-Injection Differential-Pulse Anodic Stripping Voltammetry as a Tool for Thallium Monitoring in the Environment. Electroanalysis.

[56] John Peter A.L., Viraraghavan T. (2005). Thallium: A review of public health and environmental concerns. Environ. Int..

[57] Leung K.M., Ooi V.E.C. (2000). Studies on thallium toxicity, its tissue distribution and histopathological effects in rats. Chemosphere.

[58] Spano N., Panzanelli A., Piu P.C., Pilo M.I., Sanna G., Seeber R., Tapparo A. (2005). Anodic stripping voltammetric determination of traces and ultratraces of thallium at a graphite microelectrode Method development and application to environmental waters. Anal. Chim. Acta.

[59] Sabbioni E., Minoia C., Ronchi A., Hansen B.G., Pietra R., Balducci C. (1994). Trace Element Reference Vlues in Tissues from Inhabitants of The European Union. VIII Thallium in the Italian Population. Sci. Total Environ..

[60] Emsley J. (2006). Thallium. The Elements of Murder: A History of Poison.

[61] Nordberg G.F., Fowler B.A., Nordberg M., Friberg L. (2007). Handbook on the Toxicology of Metals.

[62] Gareth J., Witten D., Hastie T., Tibshirani R. (2015). An Introduction to Statistical Learning: With Applications in R. Book.

[63] Murray L. (2010). Biostatistical Design and Analysis Using R: A Practical Guide.

[64] Everitt B.S. (2011). Cluster Analysis.

[65] Montgomery D.C., Runger G.C. (2011). Applied Statistics and Probability for Engineers.

[66] Scheffe H. (1999). The Analysis of Variance.

[67] Ward J.H. (1963). Hierarchical Grouping to Optimize an Objective Function. J. Am. Stat. Assoc..

[68] Rousseeuw P.J. (1987). Silhouettes: A Graphical Aid to the Interpretation and Validation of Cluster Analysis. J. Comput. Appl. Math..

[69] R Core Team (2019). R: A Language and Environment for Statistical Computing.

[70] Kassambara A., Mundt F. Factoextra: Extract and Visualize the Results of Multivariate Data Analyses (1.0.4 ed.) R Package. http://www.sthda.com/english/rpkgs/factoextra.

[71] Hadley W. (2016). Ggplot2: Elegant Graphics for Data Analysis.

[72] Hadley W. (2017). Tidyverse: Easily Install and Load the ‘Tidyverse’. https://CRAN.R-project.org/package=tidyverse.

[73] Xie Y. (2015). Dynamic Documents with R and Knitr.

